# Outcomes of reconstructive endovascular treatment of vertebrobasilar dissecting aneurysms with intramural hematoma

**DOI:** 10.3389/fneur.2022.914878

**Published:** 2022-08-11

**Authors:** Yisen Zhang, Qichen Peng, Yangyang Zhou, Chao Wang, Longhui Zhang, Xinjian Yang, Shiqing Mu

**Affiliations:** Department of Interventional Neuroradiology, Beijing Neurosurgical Institute, Beijing Tiantan Hospital, Capital Medical University, Beijing, China

**Keywords:** intracranial aneurysm, dissection, intramural hematoma, vertebrobasilar artery, endovascular treatment

## Abstract

**Background:**

Vertebrobasilar dissecting aneurysms (VBDAs) with an intramural hematoma (IMH) usually cause symptoms because of mass effect and grow in size over time. Clinical outcomes are generally poor.

**Objective:**

This study aimed to examine outcomes of reconstructive endovascular treatment (EVT) in patients with VBDAs with IMH. Safety and effectiveness were compared between flow diverters (FDs) and conventional stents.

**Methods:**

We retrospectively analyzed the clinical and radiological data of 36 VBDAs with IMH in 36 patients who underwent EVT with either FDs or conventional stents from January 2012 to December 2020 at our institution.

**Results:**

Among the 36 study patients, 20 were treated with FDs and 16 with conventional stents. Incidence of procedure-related complications did not significantly differ between the two stents. IMH growth occurred after EVT in a significantly higher proportion of conventional stent group aneurysms (zero vs. 31.3% [5/16]; *p* = 0.012). Among the five aneurysms with IMHs that grew, all recurred. Change in IMH size after EVT was significantly lower in the FD group (−2.7 vs. +8.1%, p = 0.036). However, after the recurrent aneurysms were removed from the conventional stent group, change in IMH size did not significantly differ between the two groups (−2.7 vs. +1.0%, *p* = 0.332). The proportion of patients who experienced an improvement in mRS score after EVT was significantly higher in the FD group (60 vs. 25%, *p* = 0.036).

**Conclusion:**

IMHs in VBDAs stop growing after successful reconstructive EVT. Although both FD and conventional stent treatment are effective, FD treatment may be superior based on clinical outcomes and effect on IMH size.

## Introduction

Spontaneous intracranial vertebrobasilar dissecting aneurysm (VBDA) is an important cause of subarachnoid hemorrhage (SAH) and posterior circulation ischemic stroke in young and middle-aged adults (1, 2). Digital subtraction angiography (DSA) is the gold standard for VBDA diagnosis and follow-up; however, it is associated with complications such as iatrogenic arterial dissection and is relatively limited in showing arterial wall characteristics such as intramural hematoma (IMH) (3). In contrast, magnetic resonance imaging (MRI) can depict IMH and other findings associated with dissection (4, 5). IMH appears to be crucial for dissection progression and generation of symptoms (6).

At present, there are two main hypotheses regarding IMH formation. One theory stipulates that the hematoma begins as circulating blood enters the arterial wall after sudden disruption of both the inner elastic plate and tunica media; another hypothesizes that it originates from the vasa vasorum (7). Aneurysms with IMH will continue to progress, which can be neurologically devastating or even fatal. Although surgery for these aneurysms is theoretically more effective, the operation is high-risk and technically difficult (6, 8). Endovascular treatment (EVT) is generally considered the first-line option in VBDA management; however, its effect on IMH growth in these aneurysms remains unclear (9). This study aimed to examine outcomes in patients with VBDAs with IMH who underwent reconstructive EVT. We also aimed to compare safety and effectiveness between treatment with flow diverters (FDs) and treatment with conventional stents.

## Materials and methods

### Patient selection and data collection

**Figure 1:**
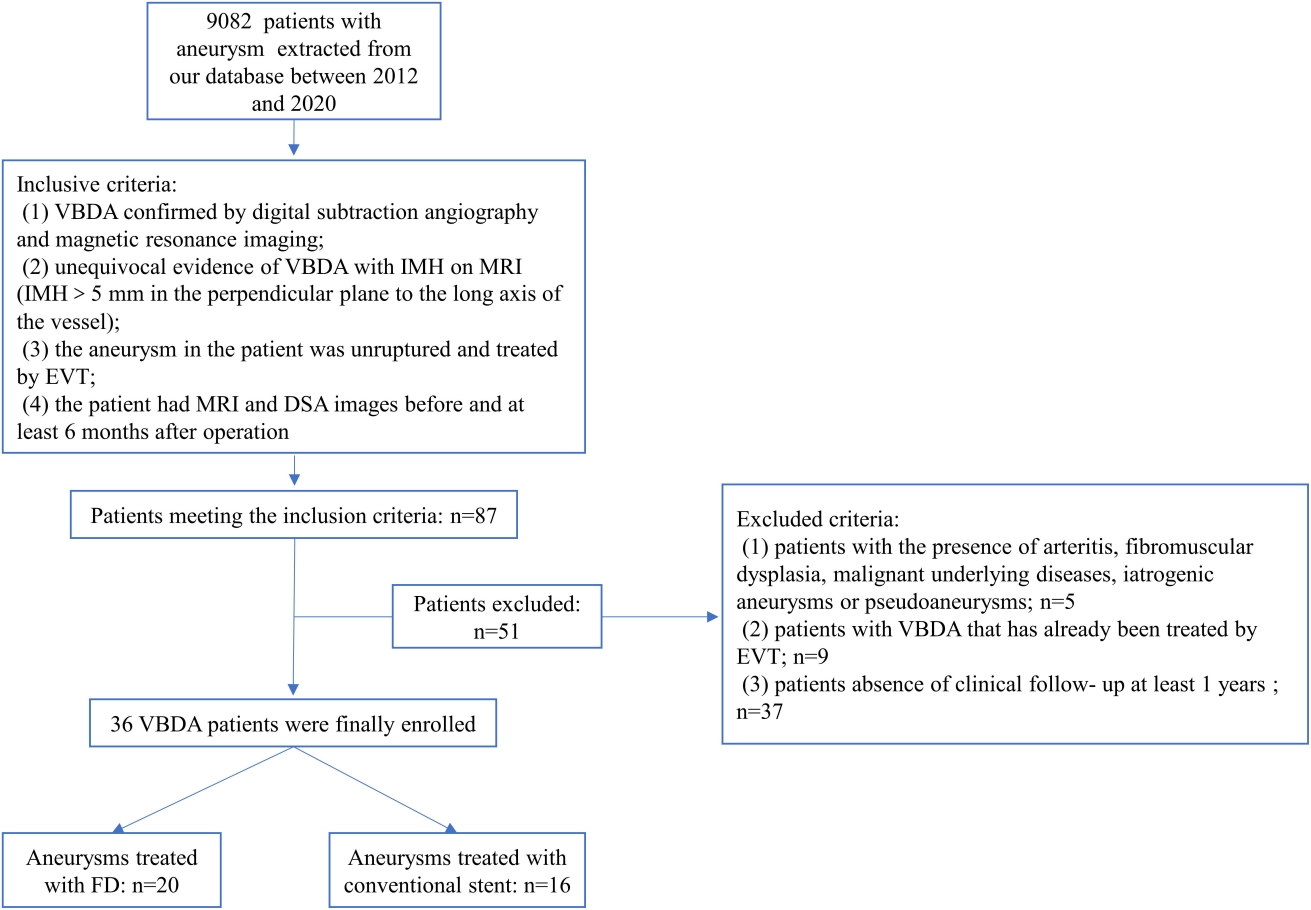
Study flowchart.

This retrospective study was approved by the ethics committee of Beijing Tiantan Hospital. We searched our aneurysm database, which includes patients diagnosed with intracranial aneurysms between January 2012 and December 2020, and identified patients with VBDA with IMH. Patients who met the following criteria were eligible for study inclusion: (1) VBDA confirmed by DSA and MRI; (2) unequivocal evidence of IMH on MRI (IMH >5 mm in the plane perpendicular to the long axis of the vessel); (10) (3) EVT was performed; and (4) follow-up MRI was performed at least 6 months after treatment. We excluded patients with arteritis, fibromuscular dysplasia, underlying malignancy, iatrogenic aneurysm, pseudoaneurysm, or VBDA that had been previously treated. We also excluded those who had no clinical follow-up. The study flow chart is shown in Figure 1. Hospital records and radiological studies were reviewed. Recorded data included patient age, sex, comorbidities, smoking and alcohol history, and symptoms; treatment strategy; modified Rankin scale (mRS) score at presentation, discharge and follow-up; and size of aneurysm and IMH. IMH size was defined as the maximum diameter of the IMH on axial MRI.

### Endovascular treatment strategy

Treatment for each patient was discussed and rendered after consensus was reached among the neurointerventionalists at the daily peer-reviewed endovascular conference in our hospital. Decisions were based on imaging parameters and clinical symptoms. Patients scheduled for reconstructive treatment received dual antiplatelet therapy (75 mg of clopidogrel and 100 mg of aspirin daily) for 5 days before treatment. Endovascular procedures were performed under general anesthesia. Full heparinization was used during the procedures to maintain an activated clotting time 2.5 times greater than baseline. In the early stage of the study, FDs were not available in our hospital. During that period, we preferred to place coils in the aneurysmal lumen after conventional stent placement if possible; if there was not sufficient space in the lumen for coiling, we treated with conventional stenting alone. Multiple devices were used in patients with long lesions or large aneurysms. The following conventional stents were used: Enterprise (Cerenovus, Raynham, Massachusetts, USA), LVIS (MicroVention, Tustin, California, USA), Neuroform EZ (Stryker Neurovascular, Fremont, California, USA), and Solitaire (Covidien, Irvine, California, USA). The stent delivery catheter was placed distal to the dissection and a microcatheter was placed within the VBDA. After deploying coils, the stent was released. The FD used was the Pipeline embolization device (Medtronic, Minneapolis, MN, USA). In patients who underwent FD placement, a microcatheter was placed into the aneurysm lumen for coil placement under microwire guidance. A triaxial support system was used to access the aneurysm with the FD introduced through a Marksman microcatheter (EV3, Irvine, California, USA). FDs were delivered to satisfactorily reconstruct the parent artery and then deployed. In patients undergoing treatment with FDs, we used the stent–jailing technique to coil the aneurysm or eccentric lumen if the diameter of the aneurysm or eccentric lumen exceeded 10 mm. One side of the vertebral artery was occluded to reduce aneurysm flow and avoid postoperative bleeding for giant vertebrobasilar junction aneurysms. After EVT, patients in the conventional stent group received 75 mg clopidogrel daily for 6 weeks and 100 mg aspirin daily for 6 months; those in the FD group received clopidogrel for 3 months and will continue taking aspirin for life.

### Follow-up and clinical outcomes

Patient data was obtained from hospital and outpatient records and *via* telephone. Clinical outcome was measured using the mRS score. Favorable clinical outcome was defined as mRS score 0–2; poor clinical outcome was defined as mRS score 4–6. Angiographic results were determined immediately after the procedure and during follow-up. DSA follow-up was scheduled between 3 and 6 months after EVT. Results were classified using the O'Kelly–Marotta (OKM) grading scale (A, total filling; B, subtotal filling; C, entry remnant; D, no filling). Favorable angiographic outcome was defined as OKM grades C and D; unfavorable angiographic outcome was defined as OKM grades A and B. Aneurysm recurrence during follow-up was defined as an increase in contrast filling within the aneurysm. MRI follow-up was scheduled 1, 2, and 5 years after EVT. A >10% increase in IMH size was defined as IMH growth (a change <10% may be due to manual error or imaging artifact).

### Statistical analysis

Statistical analyses were performed using SPSS software version 25 (IBM Corp., Armonk, NY, USA). Continuous variables are presented as means with standard deviation. Categorical variables are reported as proportions. The Shapiro–Wilk test was used to assess normality of variables. Patients and aneurysms were grouped according to type of treatment (conventional or FD stent). Group comparisons were performed using the independent samples *t-*test, χ^2^ test, or Fisher exact test as apprpriate. *P* <0.05 was considered significant.

## Results

### Patient characteristics

In total, 36 VBDAs with IMH in 36 patients who underwent reconstructive EVT were included for analysis. All patients were symptomatic at the time of treatment and all aneurysms were unruptured. Twenty were treated with FDs and 16 with conventional stents. Mean patient age in the FD and conventional stent groups was 47.1 and 55.8 years, respectively; the difference was not significant. Similarly, the groups did not significantly differ in terms of other baseline characteristics. One patient in each group had an unfavorable mRS score at admission: the FD group patient presented with left limb weakness, facial asymmetry and weakness, adverse speech, and tinnitus with mRS score 4; the conventional stent group patient presented with dizziness and unstable gait with mRS score 3. Patient and aneurysm characteristics are shown in Table 1.

**Table 1:** Patient and aneurysm characteristics.

	**FD group**	**Conventional** **stents group**	**Significance** **(*P*-Value)**
Patients	20	16	
Mean age (yrs)	47.1 ± 16.7	55.8 ± 7.3	0.063
Female, *n*(%)	5 (25%)	1 (6.3%)	0.147
**Co-morbidities**, ***n*****(%)**			
Hypertension	10 (50%)	11 (68.8%)	0.32
Diabetes	2 (10%)	1 (6.3%)	1
Smoking	8 (40%)	7 (43.8%)	1
Drinking	6 (30%)	3 (18.8%)	0.7
**Presentation**, ***n*****(%)**			
Headache	9 (45%)	9 (56.2%)	
Dizziness	3 (15%)	3 (18.8%)	
Brainstem compression	5 (25%)	2 (12.5%)	
Stroke	3 (15%)	2 (12.5%)	
Mean aneurysm diameter	18.4 ± 8.3	17.3 ± 7.0	0.697
**Aneurysm size**, ***n*****(%)**			0.935
Small (<10 mm)	2 (10.5%)	2 (12.5%)	
Large (10-25 mm)	13 (68.4%)	10 (62.5%)	
Giant (>25 mm)	4 (21.1%)	4 (25%)	
**Location**, ***n*****(%)**			0.702
BA	2 (10%)	2 (12.5%)	
VBA	3 (15%)	1 (6.3%)	
VA	15 (75%)	13 (81.3%)	
Mean IMH size (mm)	15.5 ± 7.3	17.9 ± 10.0	0.406

FD, flow diverter; BA, basilar artery; VBA, vertebrobasilar artery; VA, vertebral artery; IMH, intramural hematoma.

### Postprocedural angiographic and clinical results

EVT was successful in all patients. Thirteen patients (65%) in the FD group were treated with stenting alone and seven (35%) with stent-assisted coiling. In the conventional stent group, four patients (25%) were treated with stenting alone and 12 (75%) with stent-assisted coiling. The difference in type of EVT between groups was significant (*p* = 0.023). In the FD group, 17 patients (85%) were treated with one stent, compared with only five patients (31.3%) in the conventional stent group (p = 0.002). On immediate postoperative angiography, the rate of favorable angiographic outcome (OKM grades C and D) was significantly lower in the FD group than the conventional stent group (15 vs. 66.7%, *p* = 0.004). At hospital discharge, no patient in the FD group had a poor clinical outcome; one conventional stent patient did.

Procedure-related complications occurred in three FD group patients (15%) and one conventional stent group patient (6.3%); however, the difference was not significant (*p* = 0.613). The complications were two hemorrhages and one ischemic event in the FD group and one hemorrhage in the conventional stent group. Angiographic follow-up was available in all patients. Mean angiographic follow-up was 9.7 months in the FD group and 14.3 months in the conventional stent group. At last follow-up, the proportion of patients who achieved favorable angiographic outcome (OKM grades C and D) was higher in the FD group than the conventional stent group, but the difference was not significant (86.7 vs. 71.4%, *p* = 0.390). Clinical follow-up was available in all patients. Mean clinical follow-up was 42 months in the FD group and 51 months in the conventional stent group. Favorable clinical outcome (mRS score 0–2) was achieved at last follow-up in 18 patients (90%) in the FD group and 13 patients (81.3%) in the conventional stent group (*p* = 0.637). The proportion of patients who experienced an improvement in mRS score after EVT was significantly higher in the FD group (60 vs. 25%, *p* = 0.036). Angiographic and clinical outcomes are shown in Table 2.

**Table 2:** Angiographic and clinical outcomes.

	**FD group**	**Conventional** **stents group**	**Significance** **(*P*-Value)**
Complication, *n*(%)	3 (15%)	1 (6.3%)	0.613
Clinical follow-up time (Mean, months)	42.1	51.3	0.063
MRI follow-up time (Mean, months)	24.4	24.7	0.958
Angiographic follow-up time (Mean, months)	9.7	14.3	0.198
**Treatment modality**, ***n*****(%)**			**0.041**
Stents alone	13 (65%)	4 (25%)	
Stents with coils	7 (35%)	12 (75%)	
**Number of stents implanted**, ***n*****(%)**			
1	17 (85%)	5 (31.3%)	**0.002**
2	2 (10%)	6 (37.5%)	0.103
3	1 (5%)	5 (31.3%)	0.069
**Immediate angiographic**	20	16	**0.004**
Favorable results, *n*(%)	2 (15%)	10 (66.7%)	
Unfavorable results, *n*(%)	17 (85%)	5 (33.3%)	
**Last angiographic**	15	14	0.390
Favorable results, *n*(%)	13 (86.7%)	10 (71.4%)	
Unfavorable results, *n*(%)	2 (13.3%)	4 (28.6%)	
Recurrence, *n*(%)	0 (0%)	5 (31.3)	**0.044**
Change of IMH size, *n*(%)	−2.7%	8.1%	**0.036**
**Follow-up of clinical outcome**	20	16	0.637
Favorable results, *n*(%)	18 (90%)	13 (81.3%)	
Unfavorable results, *n*(%)	2 (10%)	3 (18.8%)	
**Change in mRS score**, ***n*****(%)**			
Improved	12 (60%)	4 (25%)	**0.036**
No change	6 (30%)	8 (50%)	0.307
Worsened	2 (10%)	4 (25%)	0.374

FD, flow diverter; IMH, intramural hematoma; mRS, modified Rankin scale.

Boldface type indicates statistical significance.

### Description of change in IMH size

**Figure 2:**
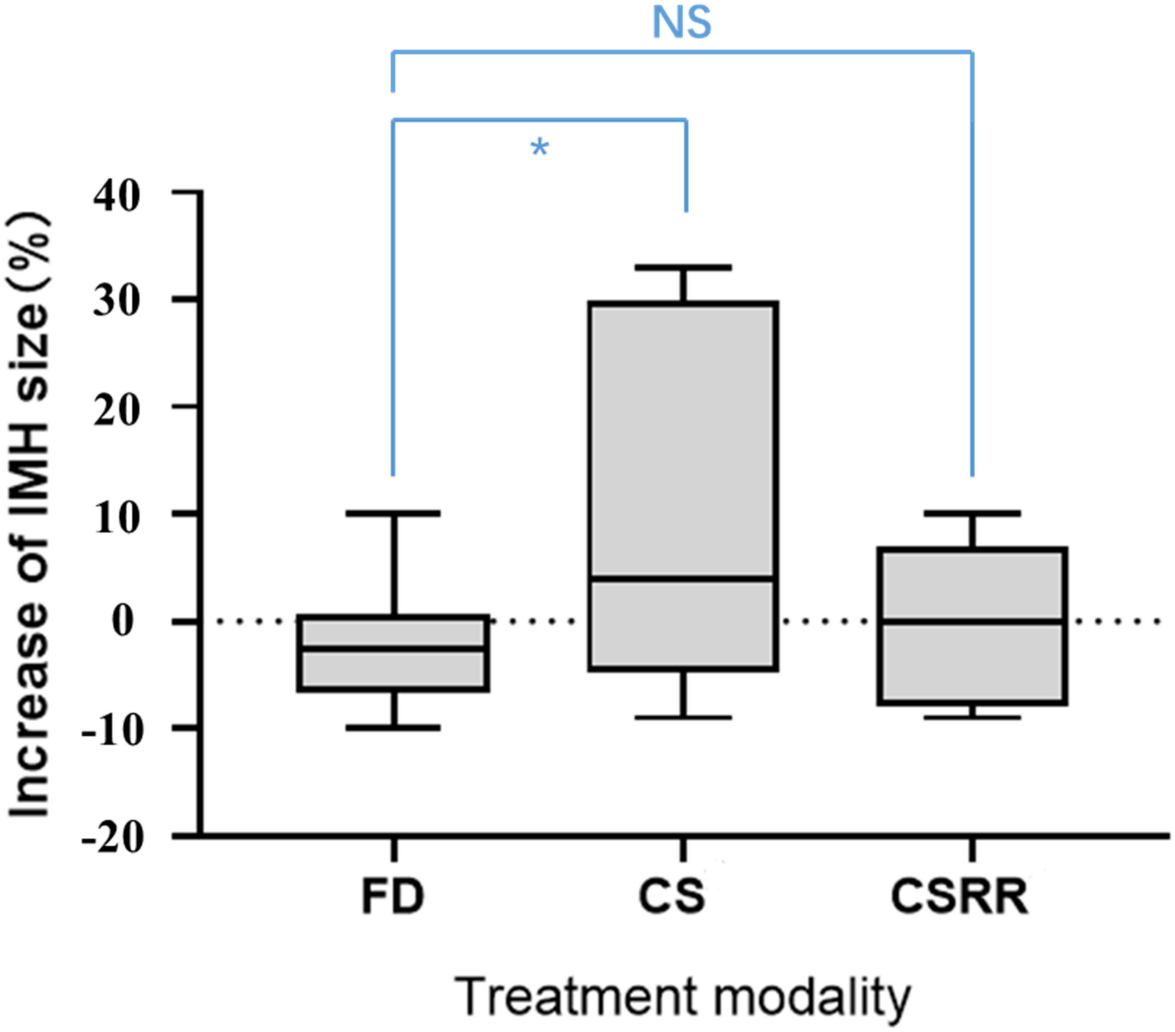
Increase in intramural hematoma size according to treatment group. CS, conventional stent group; CSRR, conventional stent group excluding recurrent aneurysms; NS, no significant difference; ^*^significant difference.

Before EVT, IMH size did not significantly differ between the FD and conventional stent groups (15.5 mm vs. 17.9 mm; *p* = 0.406). Mean MRI follow-up was 24.5 months (range, 3–80). IMH growth occurred after EVT in a significantly higher proportion of conventional stent group aneurysms (zero vs. 31.3% [5/16], *p* = 0.012). Among the five aneurysms with IMHs that grew, initial IMH size was >20 mm and all recurred after treatment. Change in IMH size after treatment was significantly lower in the FD group than the conventional stent group (−2.7% vs. +8.1%, *p* = 0.036). However, after the recurrent aneurysms were removed from the conventional stent group, change in IMH size did not significantly differ between the two groups (−2.7 vs. +1.0%, *p* = 0.332; Figure 2).

### Illustrative cases

#### Case 1

**Figure 3:**
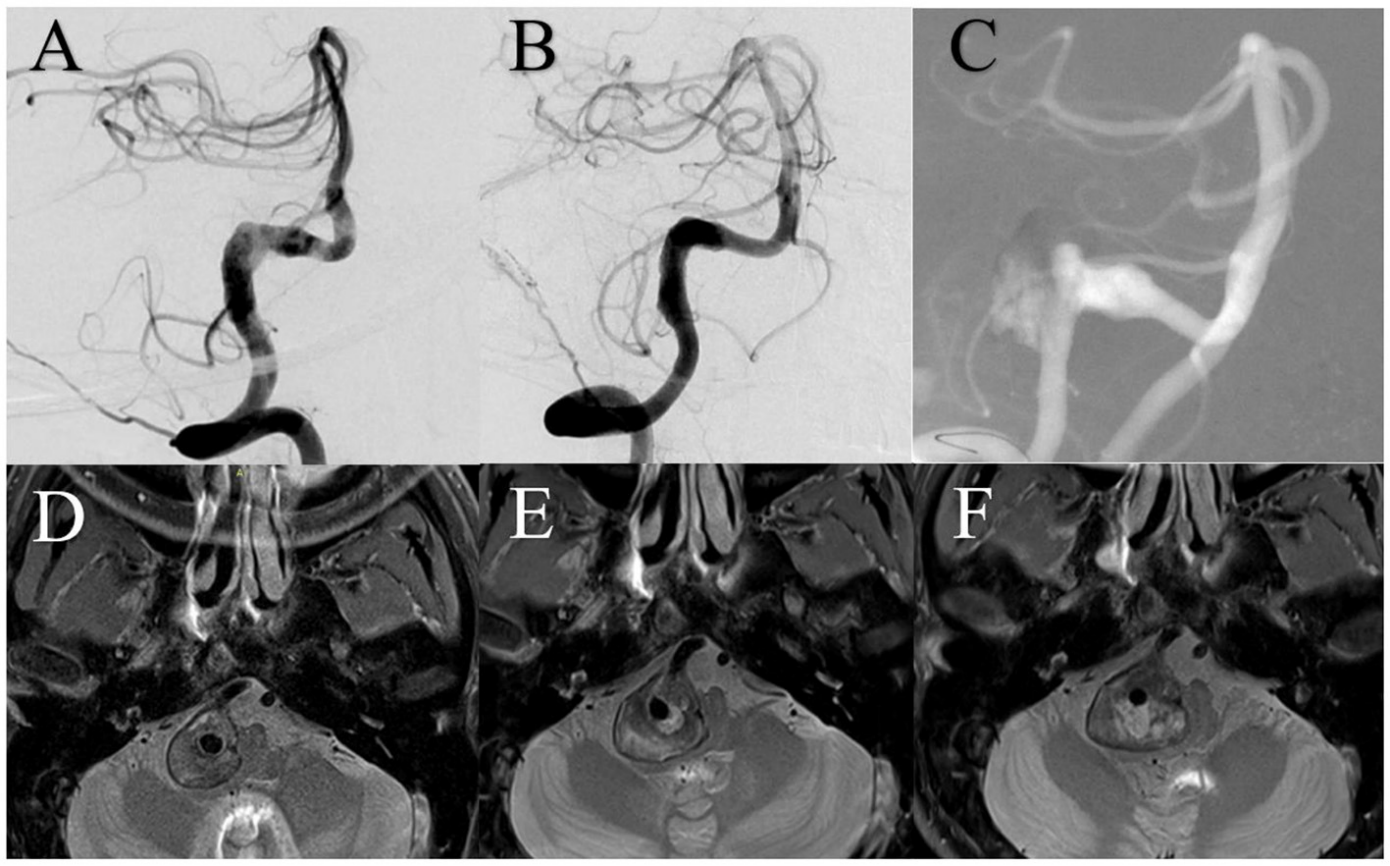
**(A)** Digital subtraction angiography (DSA) showed the right vertebral artery aneurysm. **(B)** DSA immediately after treatment showed satisfactory arterial reconstruction. **(C)** DSA 2 years after treatment showed aneurysm recurrence. Magnetic resonance imaging demonstrated intramural hematoma growth from before treatment **(D)** to 1 year **(E)** and 2 years **(F)** after.

A patient presented with a 6-month history of headaches. DSA showed a giant right vertebral artery dissecting aneurysm. MRI showed a 20.8 mm IMH. The patient was treated using three 4.5 mm × 37 mm Enterprise stents overlap without complication. Immediately after treatment, angiography showed satisfactory reconstruction of the vertebral artery and the patient's headache had improved. Two years after treatment, DSA showed aneurysm recurrence and MRI showed a 27 mm IMH, which had increased from 24.2 mm 1 year prior (Figure 3).

#### Case 2

**Figure 4:**
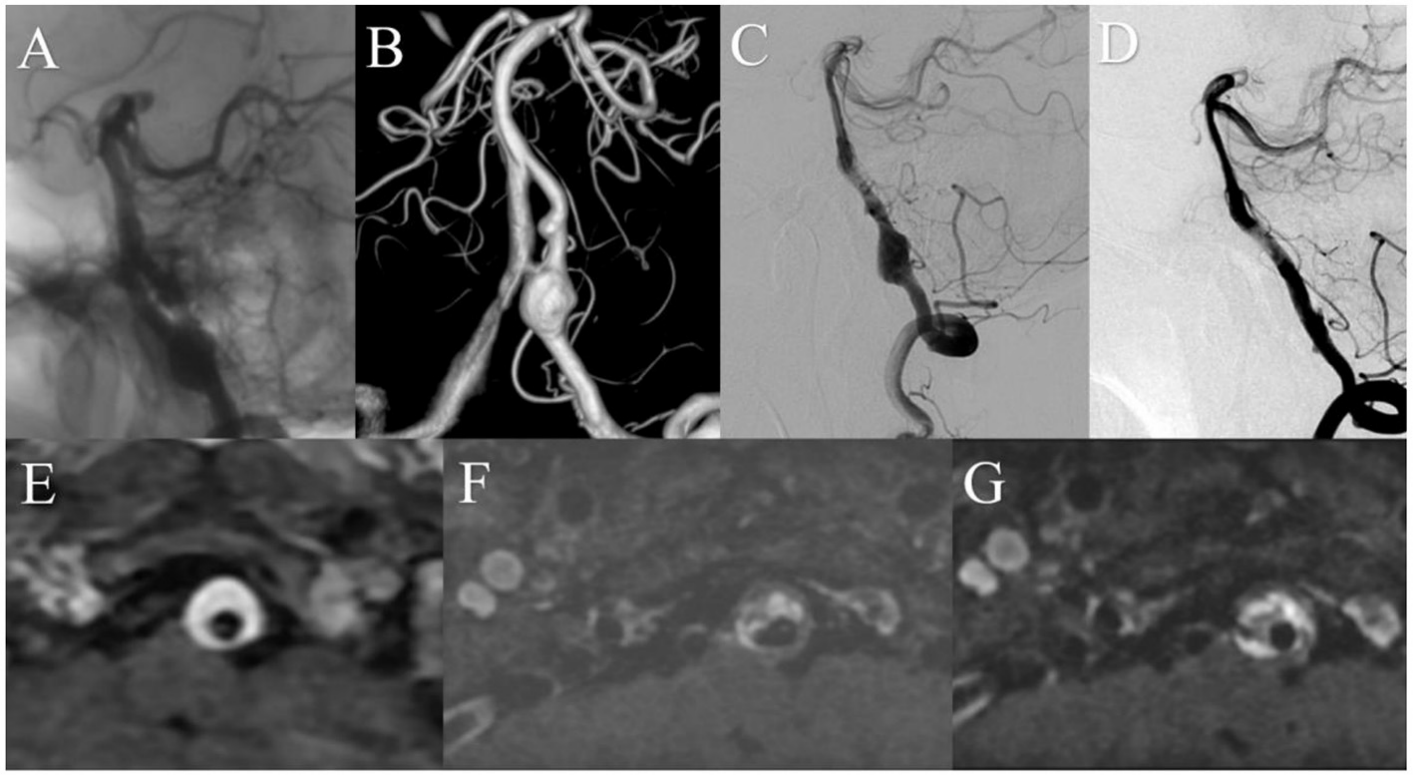
Preoperative anteroposterior **(A)** and three-dimensional reconstruction **(B)** digital subtraction angiography (DSA) showed a left vertebral artery aneurysm. **(C)** DSA immediately after treatment showed that contrast stasis within the aneurysm. **(D)** One year after treatment, DSA showed satisfactory arterial reconstruction and complete aneurysm obliteration. Magnetic resonance imaging demonstrated stability of the intramural hematoma over time [**(E)** before treatment; **(F)** 1 year after; **(G)** 3 years after].

A patient presented with a 10-month history of dizziness. DSA showed a large right vertebral artery dissecting aneurysm. MRI showed a 12.3 mm IMH. The patient was treated with a 4.5 mm × 35 mm Pipeline embolization device without complication. Immediately after treatment, angiography showed contrast stasis within the aneurysm and the patient reported symptom relief. One-year after treatment, DSA showed satisfactory arterial reconstruction and complete aneurysm obliteration. MRI showed no change in IMH size over 3 years of follow-up (Figure 4).

## Discussion

### Key results

This study examined a series of patients with VBDAs with IMH who underwent reconstructive EVT using FDs or conventional stents. In the FD group, the aneurysm recurrence rate was lower and the proportion of patients who experienced improvement in mRS score after treatment was higher than those in the conventional stent group. More importantly, IMH size continued to increase after conventional stent treatment in five patients and the aneurysm in all of these patients recurred. In both the FD and conventional stent groups, the IMH in aneurysms that did not recur stopped growing. Therefore, angiographic aneurysmal occlusion after reconstructive EVT may impair or prevent IMH growth.

### Natural history of the IMH in VBDAs

IMH usually results from extensive damage to the internal elastic lamina, rupture of neovessels, or penetration of blood into the vessel wall (11). Despite numerous pathological studies, the mechanism of aneurysmal IMH growth remains unknown. Growth may be related to bleeding from the vasa vasorum (VV), parent arterial inflow, and/or inflammation. Krings et al. suggested a mechanism of repeated subadventitial hemorrhage from the VV (12). The VV are composed of small arteries, capillaries, and veins that supply the walls of large vessels and serve as a conduit for macrophages and inflammatory, angiogenic, and other factors (13, 14). One postmortem study suggested that the VV are more developed in vessels with a thick wall to meet their higher metabolic needs; the same study also reported that approximately half of disease-free intracranial arteries have VV and that they are frequently found in the vertebrobasilar artery (15), which may explain why IMHs are common in VBDAs.

Nagahiro et al. reported different findings: their examination of VBDAs with large IMH showed no evidence of hemorrhage around the vessels in the aneurysmal wall; however, intrathrombotic vascular channels were observed. Therefore, blood flow between the parent artery and the intrathrombotic vascular channels may explain continuous IMH growth (16). This agrees with prior studies that found recanalizing vessels within the thrombus (6). Yasui et al. reported similar findings of numerous clefts in old thrombus near the wall of the distal aneurysmal neck that seemed to connect the parent artery lumen with the most peripheral fresh hemorrhage (17). Ferracci et al. also reported that parent arterial inflow rather than VV may be the cause of IMH and that shear stress on the edge of the aneurysm neck or at the vessel dissection point might drive dissection, leading to recurrent intramural hemorrhage (18). Furthermore, inflammation is involved in thrombus organization, vessel dissection, and neovascularization (12, 19, 20).

In our study, IMH growth stopped after successful reconstructive EVT but continued in aneurysms that recurred. This supports the hypothesis that parent arterial inflow contributes to IMH growth. Vascular remodeling may block parent arterial inflow; however, blood from the aneurysmal neck can penetrate into the vessel wall when aneurysms recanalize.

### Treatment strategy for VBDAs with IMH

Aneurysms with IMH progress without exception in a relatively short time, which usually leads to a poor outcome (6). Considering the potentially fatal consequences, early intervention is necessary. Before the development of EVT, surgical treatment was the mainstay. Drake et al. reported outcomes in 56 surgically treated patients with fusiform posterior circulation aneurysms. Treatments included aneurysm clipping, wrapping, or proximal ligation with or without bypass surgery. Thirteen died and four had severe neurologic deficits (21). Considering the high morbidity and mortality of surgical treatment, EVT has become widely preferred. The introduction of FDs has enabled endovascular reconstruction treatment and vascular remodeling for complex dissecting aneurysms with IMH. Treatment with FDs results in a high complete aneurysm occlusion rate. However, limited information is available regarding IMH outcome after reconstructive EVT. Moreover, the safety and effectiveness of FDs in the treatment of these aneurysms is unclear and has not been compared with conventional treatment.

We found similar favorable clinical outcome rates in patients treated using FDs and conventional stents (90 and 81.3%, respectively; *p* = 0.637). This is in line with favorable outcome rates of 85.7 and 92.0% reported in two recent studies of reconstructive EVT for VBDAs (22, 23). Considering that all aneurysms in our study had a large IMH, the efficacy of both FDs and conventional stents was acceptable. The rate of favorable angiographic outcome was significantly lower in the FD group than the conventional stent group immediately after treatment (15 vs. 66.7%, *p* = 0.004); however, at last angiographic follow-up, the same rate was actually higher in the FD group (86.7 vs. 71.4%, *p* = 0.390) but the difference was not significant, possibly because of the small sample size. This finding agrees with prior studies that reported higher long-term occlusion rates in the FD group (24). In addition, we found that the recurrence rate was higher in the conventional stent group (zero vs. 31.3%, p = 0.044). Jeon et al. (25) studied 47 patients with VBDAs who underwent stent-assisted coil embolization with conventional stents; recurrence occurred in 10 (21.2%). In another study of posterior circulation aneurysms treated with FDs, the retreatment rate was 8.4% (26).

Compared to conventional stenting (even with adjunctive coiling), FD treatment is superior in terms of the long-term occlusion rate, primarily because FDs completely seal the aneurysm neck and divert flow away from the aneurysm, which leads to aneurysmal thrombosis and shrinkage (27, 28). The major concern with use of FDs for posterior circulation aneurysms is their high complication rate. Nonetheless, some studies have shown favorable outcomes. Zhang et al. (29) compared the incidence of complication between FD and stent-assisted coiling treatment of unruptured posterior circulation non-saccular aneurysms; the two groups did not differ in terms of periprocedural complications, technical events, or delayed complications. In a study of large or giant non-saccular vertebrobasilar aneurysms, similar results were obtained (24). Natarajan et al. (30) reported 11 patients with posterior circulation aneurysms who underwent FD treatment; only one experienced a perforator stroke while the others had a good outcome. They suggested that flow diversion is evolving to become a safer treatment option.

VBDAs with IMH usually present with progressive mass effect because of IMH growth. The true lumen may become more stenotic in the presence of an IMH, which may lead to embolic ischemic events (10, 31, 32). Therefore, recurrence of these aneurysms may be more dangerous. Our study also illustrates this point: five patients treated with conventional stenting experienced aneurysm recurrence. The IMH continued to grow in all five and two of them died. Considering the lower recurrence rate and better symptom improvement in the patients treated with FDs, early FD treatment of these aneurysms should be highly considered.

### Hypothesis of IMH outcome for symptom improvement

In the present study, the proportion of patients who experienced improvement in mRS score at last follow-up was significantly higher in the FD group (60% vs. 25%, *p* = 0.036). This result is not unexpected considering that previous studies have reported that the rate of symptom improvement is high in patients with dissecting posterior circulation aneurysms after FD treatment (26, 33). Our results also suggest that IMH size did not increase in patients with aneurysms that did not recur. We speculate that the improvement in symptoms is attributed to several factors.

First, symptoms may improve owing to the “water-hammer effect.” In fluid dynamics, the water- hammer effect occurs when high-velocity fluid rapidly changes momentum, which erodes or destroys the surface with which it contacts. A basilar aneurysm study suggested that aneurysms with a wide neck or those that incorporate a major arterial branch are subject to constant arterial pulsations that cause motion of the IMH, which results in increased mass effect from the aneurysm (34). Tomokiyo et al. (35) suggested that a persistent water-hammer effect against the aneurysmal lumen as well as an IMH-induced increase in aneurysmal volume may contribute to the development of perianeurysmal edema. We therefore hypothesize that blood flow in the aneurysm transmits pulsations and gradually aggravates neurological symptoms because of the development of mass effect and perianeurysmal edema. After reconstructive EVT, pulsations decrease and the IMH stops growing, which alleviates mass effect and perianeurysmal edema and improves patient symptoms. Furthermore, FDs can promote endothelialization of the aneurysm neck and combat the water-hammer forces (36).

Inflammation may be another factor related to symptom improvement. Suzuki et al. (37) suggested that microvascularization owing to microbleeds and inflammation from microvessels occur in a vicious cycle, which causes neurological symptoms. Moreover, repeated hemodynamic insults after dissecting aneurysm formation leads to periods of inflammation and thrombosis, which exacerbates this cascade. However, with reconstructive EVT, the aneurysmal neck is completely covered by a layer of long slender cells resembling endothelium three to 12 months after treatment and there is little inflammatory cellular reaction in the aneurysm dome (38). Therefore, early EVT may reduce patient symptoms. As mentioned above, FDs may be a better choice than conventional stents because they are superior at promoting endothelialization of the aneurysm neck and eliminating intra-aneurysmal inflammation. However, our speculations regarding IMH size and clinical improvement require further study.

### Limitations

Our study has several limitations. It is retrospective in nature and was conducted in a single center. Given the rarity of VBDAs with IMH, our sample size was small. In addition, the study time period was long, during which technical nuances of treatment changed. Therefore, both selection and treatment bias may have been introduced. Moreover, the follow-up period was short and MRI measurements of IMH may have been affected by manual errors and imaging artifact from metal devices (coils or stent).

## Conclusion

IMHs in VBDAs stop growing after successful reconstructive EVT but continue to grow in aneurysms that recur. Successful vascular remodeling may block penetration of parent arterial flow into the aneurysm. Although both FD and conventional stent treatment are effective, FD treatment may be superior based on clinical outcomes and effect on IMH size.

## Data availability statement

The original contributions presented in the study are included in the article/supplementary material, further inquiries can be directed to the corresponding authors.

## Ethics statement

The studies involving human participants were reviewed and approved by Institutional Review Board of Beijing Tiantan Hospital. The patients/participants provided their written informed consent to participate in this study.

## Author contributions

YZha and QP collected the clinical data, performed the statistical analysis, and wrote the manuscript. YZho, CW, and LZ helped collect the clinical data. XY and SM helped revise the manuscript, designed the research, and handled funding and supervision. All authors read and approved the final manuscript.

## Funding

This work was supported by the National Natural Science Foundation of China (Grant Numbers: 81801158 and 81571128).

## Conflict of interest

The authors declare that the research was conducted in the absence of any commercial or financial relationships that could be construed as a potential conflict of interest.

## Publisher's note

All claims expressed in this article are solely those of the authors and do not necessarily represent those of their affiliated organizations, or those of the publisher, the editors and the reviewers. Any product that may be evaluated in this article, or claim that may be made by its manufacturer, is not guaranteed or endorsed by the publisher.
